# Evaluation of serum concentrations of total protein and gamma-globulin as an indicator of serum immunoglobulin G concentration in dairy calves

**DOI:** 10.3168/jdsc.2023-0469

**Published:** 2024-04-20

**Authors:** K. Murayama, N. Kobayashi, N. Nishizawa, M. Oba, T. Sugino

**Affiliations:** 1 Dairy Technology Research Institute, The National Federation of Dairy Co-operative Associations (ZEN-RAKU-REN), Nishi-shirakawa, Fukushima, Japan 969-0223; 2 The Research Center for Animal Science, Graduate School of Integrated Science for Life, Hiroshima University, Higashi-Hiroshima, Japan 739-8528; 3 Department of Agricultural, Food and Nutritional Science, University of Alberta, Edmonton, Alberta, Canada T6G 2P5

## Abstract

The aim of this study was to evaluate precision of estimating serum IgG concentration from total protein (TP) or gamma-globulin (γGLB) concentration as an alternative approach, and to compare morbidity of preweaning dairy calves differing in serum γGLB concentration. In trial 1, blood was sampled from 129 Holstein calves in the first week after birth, and serum concentrations of TP, γGLB, and IgG were measured. Spearman's correlation coefficient (r_s_) between serum IgG and TP concentrations was 0.89, and r_s_ between serum IgG and γGLB concentrations was 0.96. Absolute residual (observed − predicted) serum IgG concentrations were smaller when estimated by serum γGLB concentration than by serum TP concentration, and differences in the absolute residuals were smaller for calves fed colostrum replacer (1.68 vs. 4.29 g/L) than those fed whole colostrum (2.41 vs. 3.48 g/L). In trial 2, blood was sampled from 740 Holstein heifer calves during the first week of age, and serum γGLB concentration was measured. The calves were divided into 4 categories based on their serum γGLB concentration; ≥1.0 g/dL (excellent), 0.7 ≤ γGLB <1.0 g/dL (good), 0.4 ≤ γGLB <0.7 g/dL (fair), and <0.4 g/dL (poor). Morbidity for diarrhea and respiratory disease in preweaning dairy calves was determined for the first 28 and 56 d of age, and compared among the 4 categories based on serum γGLB concentration. Calves with serum γGLB concentration higher than 0.7 g/dL (good and excellent) had less diarrhea during the first 28 d of age than those with lower serum γGLB concentration (fair and poor). Calves with serum γGLB concentration higher than 1.0 g/dL (excellent) had less respiratory diseases for the first 56 d of age than those with lower serum γGLB concentration (good, fair, and poor). These results suggest that serum IgG concentration can be estimated more precisely from concentration of γGLB than TP particularly for calves fed colostrum replacer, and that γGLB concentrations in the first week of age are associated with morbidity of calves. Transfer of passive immunity in dairy calves can be assessed effectively by serum γGLB concentration.

## Highlights

• Serum γGLB was correlated with serum IgG concentration.
• Serum γGLB is a precise proxy for IgG in calves fed colostrum or CR.
• Serum γGLB is a predictive indicator for calf health similar to serum IgG.

## Graphical Abstract

**Figure:**
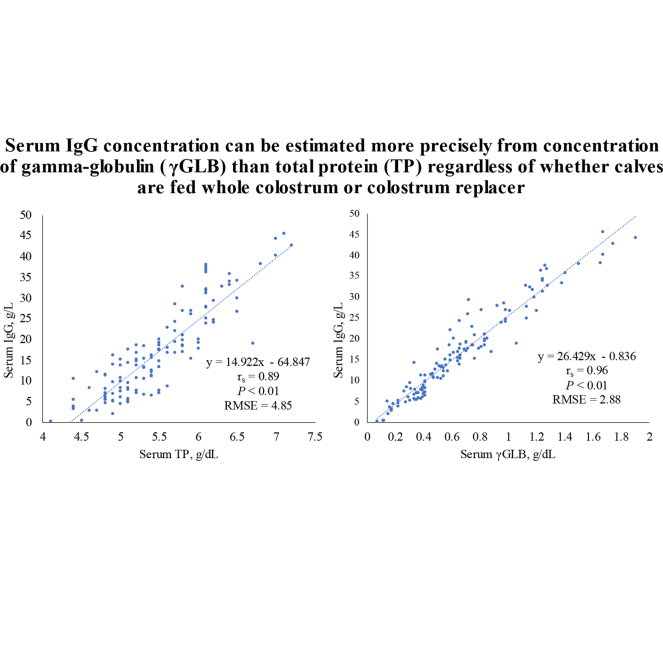
**Summary:** Serum total protein (TP) concentration has been used as an indicator of passive immunity. It works for calves fed colostrum replacer (CR) but may not be the most accurate, possibly due to variable amount, type, and manufacturing process of protein. Therefore, it is necessary to identify a biomarker that can be used to estimate serum immunoglobulin G (IgG) concentration precisely, even in calves fed CR. This study showed that serum gamma-globulin (γGLB) concentration was correlated with serum IgG concentration. In addition, absolute residual (observed - predicted) serum IgG concentrations were smaller when estimated by serum γGLB concentration than by serum TP concentration, particularly for calves fed CR. Calves with higher serum γGLB concentration had less diarrhea or respiratory diseases before weaning. These results suggest that transfer of passive immunity can be assessed effectively by serum γGLB concentration, regardless of whether calves are fed whole colostrum or CR.

Calves are born without passive immunity because the placental structure of ruminant dam prevents the transfer of maternal serum IgG to the calf (Davis and Drackley, 1998). Therefore, ingestion of colostrum is necessary for calves to acquire IgG and other immune factors or cells, and this process is known as transfer of passive immunity. Measurement of serum concentration of IgG is the gold standard method to assess transfer of passive immunity in newborn dairy calves, but its analysis is expensive and may not be cost-effective. Therefore, serum concentration of total protein (**TP**) has been used as a common indicator to assess transfer of passive immunity as it is highly correlated with serum IgG concentration in calves fed whole colostrum (**WC**) (Calloway et al., 2002; Deelen et al., 2014; Lopez et al., 2021). However, R^2^ between serum concentrations of TP and IgG is lower for calves fed colostrum replacer (**CR**) compared with those fed WC (Lopez et al., 2021). As a variety of CR products has become available lately, colostrum management has been greatly diversified among dairies. Therefore, it is necessary to identify a biomarker to estimate serum IgG concentration precisely regardless of whether calves are fed WC or CR.

Serum gamma-globulin (**γGLB**) is a protein fraction measured by electrophoresis (Bossuyt et al., 1998), and its analysis costs are generally far lower than IgG measurements as it is routinely analyzed in commercial clinical laboratories. Nonetheless, because IgG is the primary component of γGLB, while TP contains many other protein fractions, we hypothesized that serum γGLB concentration could be used to estimate serum IgG concentration more precisely than TP regardless of type of colostrum fed to newborn calves. The aim of this study was to evaluate precision of estimating serum IgG concentration from TP and γGLB as alternative approaches (trial 1), and to evaluate relationship between serum γGLB concentration and morbidity in preweaning dairy calves (trial 2).

All experimental procedures were approved by the Animal Care and Use Committee of ZEN-RAKU-REN (Fukushima, Japan). In trial 1, blood was sampled from 129 Holstein calves in the first week after birth on 33 dairies between September 22, 2019, and June 17, 2021. Seventy-four calves were fed WC, and 55 calves were fed multiple CR products at the first feeding after birth. Thirty-three calves in 55 CR fed calves were fed whey-based CR products, 13 calves were fed skim milk powder-based CR product, 6 calves were fed dried colostrum products. The type of CR products fed to 3 calves was unknown. Blood was sampled from a jugular vein during the first week of age using evacuated tubes (Insepack II SMD108CG with procoagulant and separating medium; Sekisui Medical, Tokyo, Japan), centrifuged at 1,800 × *g* at 4°C for 20 min, and serum was harvested. Serum samples were stored at −20°C, and analyzed for IgG, TP, and γGLB concentrations. Serum IgG concentration was measured by a single radial immunodiffusion method using a commercial kit (Bovine IgG SRID assay kit LL-70002, Life Laboratory, Yamagata, Japan). Serum TP and γGLB concentrations were measured by a commercial laboratory (KOTOBIKEN Medical Laboratories, Tokyo, Japan) using the biuret method and cellulose acetate membrane electrophoresis, respectively. Two linear regression equations were developed to estimate serum concentration of IgG from TP or γGLB concentration, and Spearman's correlation coefficients (**r_s_**) were determined for each model using JMP Pro 16 (SAS Institute Inc., Cary, NC). Sensitivity was defined as the probability that the estimated IgG value is ≥10 g/L for samples with IgG ≥10 g/L. Specificity was defined as the probability that the estimated IgG value is <10 g/L for samples with IgG <10 g/L. Absolute residual (observed − predicted) serum IgG concentrations were analyzed using JMP Pro 16 (SAS Institute Inc.) with the following model:

Y_ij_ = μ + T_i_ + I_j_ + TI_ij_ + e_ij_,

where Y_ij_ is the dependent variable, μ is the overall mean, T_i_ is the fixed effect of colostrum type (WC or CR), I_j_ is the fixed effect of indicator to estimate IgG concentration (serum concentrations of TP or γGLB), TI_ij_ is the interaction between colostrum type and indicator, and e_ij_ is the residual.

In trial 2, 740 Holstein heifer calves were transported from commercial dairies to a calf nursing farm at 3 to 7 d of age; blood was sampled immediately after arrival, serum was harvested, and γGLB concentrations were measured as described for trial 1. The calves were divided into 4 categories based on their serum γGLB concentration: ≥1.0 g/dL (excellent), 0.7 ≤ γGLB <1.0 g/dL (good), 0.4 ≤ γGLB <0.7 g/dL (fair), and <0.4 g/dL (poor). These categories were developed according to Lombard et al. (2020) and the equation to estimate serum IgG concentration from γGLB. Morbidity, defined as veterinary intervention, for diarrhea or respiratory disease in preweaning dairy calves was determined for the first 28 and 56 d of age, and the 4 categories based on serum γGLB concentrations were compared in the morbidity by chi-squared test using JMP 16 Pro (SAS Institute Inc.).

In trial 1, serum IgG, TP, and γGLB concentrations were 16.8 ± 10.69 g/L, 5.5 ± 0.64 g/dL, and 0.67 ± 0.390 g/dL (mean ± SD), respectively. The range in serum IgG, TP, and γGLB concentrations were 0.3 to 45.5 g/L, 4.1 to 7.2 g/dL, and 0.07 to 1.90 g/dL, respectively. The r_s_ between serum IgG and TP concentration was 0.89 (*P* < 0.01; Figure 1A), and r_s_ between serum IgG and γGLB was 0.96 (*P* < 0.01; Figure 1B). Sensitivities for the estimation of serum concentration of IgG from TP were 0.77 and 0.58 in calves fed WC and CR, respectively. Sensitivities for the estimation of serum concentration of IgG from γGLB were 0.82 and 1.00 in calves fed WC and CR, respectively. Specificities for the estimation of serum concentration of IgG from TP were 0.96 and 0.81 in calves fed WC and CR, respectively. Specificities for the estimation of serum concentration of IgG from γGLB were 0.98 and 0.97 in calves fed WC and CR, respectively. Overall root mean squared error (**RMSE**) for the estimation of serum concentration of IgG from TP was 4.85, and RMSE in calves fed WC and CR were 4.38 and 5.34, respectively. In contrast, overall RMSE for the estimation of serum concentration of IgG from γGLB was 2.88, and RMSE in calves fed WC and CR were 3.30 and 2.11, respectively. Absolute residual (observed − predicted) serum IgG concentrations were smaller (*P* < 0.01) when they are estimated by serum γGLB concentration than by serum TP concentration. In addition, an interaction effect observed between colostrum type (WC or CR) and indicator (TP or γGLB; *P* = 0.01; Figure 2) is attributed to that the differences in absolute residuals were numerically greater in calves fed CR (4.29 vs. 1.68 g/L) than those fed WC (3.49 vs. 2.41 g/L). These results suggest that serum IgG concentrations can be estimated more precisely from concentration of γGLB than TP, particularly for calves fed CR.

**Figure 1:**
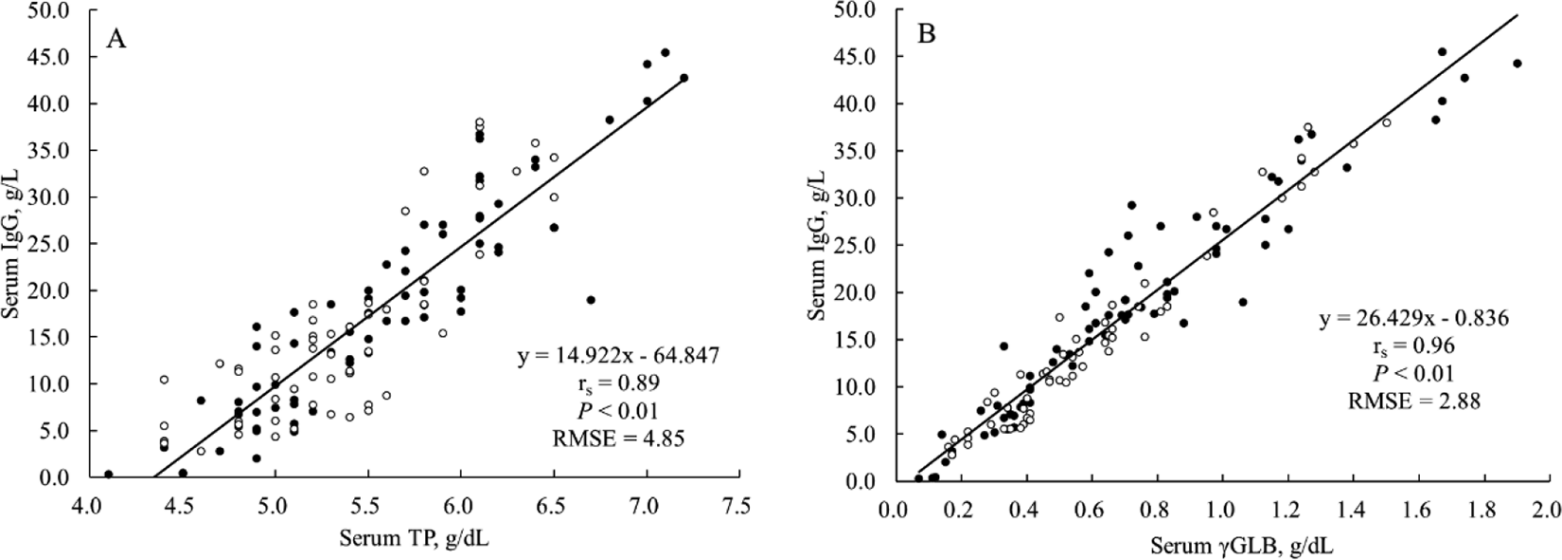
Relationships of serum IgG concentration with serum total protein (TP) concentration (A) and gamma-globulin (γGLB) concentration (B) in calves fed whole colostrum (•) or colostrum replacer (○) at the first feeding after birth.

**Figure 2:**
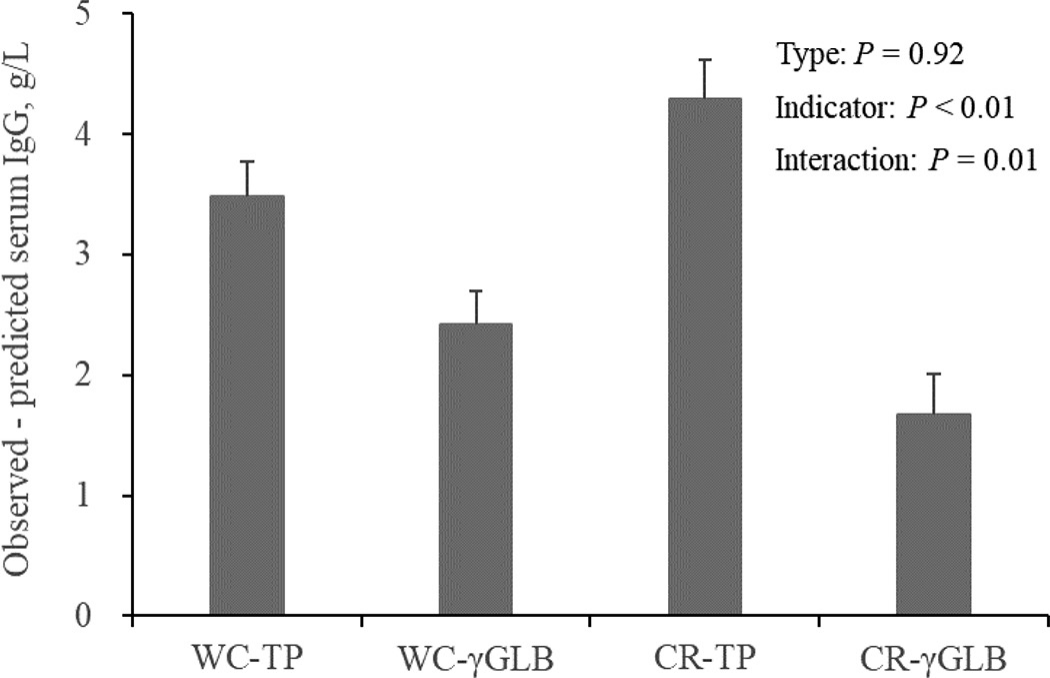
Absolute residual (observed − predicted) serum IgG concentrations estimated by total protein (TP) or gamma-globulin (γGLB) concentration in calves fed whole colostrum (WC) or colostrum replacer (CR); LSM ± SEM. Type: WC or CR. Indicator: serum concentration of TP or γGLB.

Serum TP concentration has been widely used as an indicator of passive immunity, but it may not work for calves fed CR. The lower precision in estimating IgG concentration from TP, which we found in calves fed CR, is consistent with previous findings. Mowrey (2001) reported that R^2^ between plasma concentrations of IgG and TP was lower in calves fed CR compared with those fed WC (0.62 vs. 0.72). Similarly, Lopez et al. (2021) reported that R^2^ between serum IgG and TP concentrations was 0.40 in calves fed CR, whereas it was 0.81 in calves fed WC. Serum TP includes many protein fractions such as albumin, α-1 globulin, α-2 globulin, and β-globulin as well as γGLB that includes IgG (Piza et al., 2021). Specific reasons for lower precision in estimating IgG concentration from TP concentration in calves fed CR are not known, but it may be attributed to differences in protein profile and fat content compared with WC (Lopez et al., 2021). In addition, CR calves in the current study were fed different products varying in protein amount, type, and manufacturing process, which likely led to variable curd formation, digestion, and absorption, greatly affecting serum concentrations of non-IgG proteins.

In the current study, serum TP did not appear to underestimate serum IgG concentration in calves fed CR as indicated by similar residual distribution between CR and WC calves for the entire range (Figure 1A) possibly because CR calves in the current study were fed a variety of products not only whey-based CR. Previous studies suggest that calves fed whey-based CR may not increase serum TP concentration as much as expected even when serum IgG concentrations are increased (Lopez et al., 2020; Geiger et al., 2023). Although serum TP concentration of 5.2 g/dL is commonly used as a cut-off point to define failure of transfer of passive immunity, Lopez et al. (2021) proposed that it is necessary to develop an alternate threshold of serum TP concentration for calves fed CR. However, a different threshold determined for a CR product may not work for calves fed other CR products because CR products vary in amount and type of protein (Godden et al., 2009). Considering these limitations associated with the use of serum TP, especially for calves fed CR, serum γGLB concentration would be more appropriate indicator of passive immunity regardless of type of colostrum fed to newborn calves.

In trial 2, proportions of calves categorized as excellent, good, fair, and poor, based on serum γGLB concentrations, were 18.5%, 15.8%, 33.4%, and 32.3%, respectively. Within each category, percent mortalities were 0.7%, 1.0%, 1.7%, and 3.1% for 0 to 28 d of age, and 0.7%, 1.0%, 1.7%, and 4.2% for 0 to 56 of age, respectively for excellent, good, fair, and poor (Table 1). Within each category, proportions of calves that had diarrhea were 6.6%, 10.3%, 21.1%, and 28.0% for 0 to 28 d of age, and 14.6%, 18.8%, 23.5%, and 29.7% for 0 to 56 of age, respectively, for excellent, good, fair, and poor. Calves with serum γGLB concentration higher than 0.7 g/dL (good and excellent) had less diarrhea during the first 28 d of age than those with lower serum γGLB concentration (fair and poor; *P* < 0.01). Proportions of calves that had respiratory disease were 15.3%, 21.4%, 23.5%, and 24.3% for 0 to 28 d of age, and 32.1%, 46.2%, 51.8%, and 54.0% for 0 to 56 of age, respectively for excellent, good, fair, and poor categories. Calves with serum γGLB concentration higher than 1.0 g/dL (excellent) had less respiratory diseases for the first 56 d of age than those with lower serum γGLB concentration (good, fair, and poor; *P* = 0.02). These results indicated that serum γGLB concentrations in the first week of life are associated with morbidity before weaning in dairy calves.

**Table 1:** Percent mortality and morbidity by transfer of passive immunity for Holstein calves during 0 to 56 d of age

Item	Transfer of passive immunity category^1^				*P*-value^2^		
	Excellent (n = 137)	Good (n = 117)	Fair (n = 247)	Poor (n = 239)	E vs. G	G vs. F	F vs. P
Mortality							
0 to 28 d of age	0.7	1.0	1.7	3.1	0.82	0.62	0.33
0 to 56 d of age	0.7	1.0	1.7	4.2	0.82	0.62	0.11
Morbidity							
0 to 28 d of age							
Diarrhea	6.6	10.3	21.1	28.0	0.29	<0.01	0.07
Respiratory disease	15.3	21.4	23.5	24.3	0.21	0.65	0.84
0 to 56 d of age							
Diarrhea	14.6	18.8	23.5	29.7	0.37	0.31	0.12
Respiratory disease	32.1	46.2	51.8	54.0	0.02	0.31	0.63

1 Serum γGLB; ≥1.0 g/dL (excellent), 0.7 ≤ γGLB <1.0 g/dL (good), 0.4 ≤ γGLB <0.7 g/dL (fair), and <0.4 g/dL (poor).

2 E = excellent; G = good; F = fair; P = poor.

The traditional cut-off point to determine failure of transfer of passive immunity was 10 g/L of serum IgG concentration (Calloway et al., 2002; Godden et al., 2019). This threshold was based on higher mortality rate in calves with serum IgG concentration <10.0 g/L (USDA, 1994). However, Urie et al. (2018) reported that calves with serum IgG concentration ≥15.0 g/L had lower morbidity compared with calves with less than 15.0 g/L. Therefore, Lombard et al. (2020) recommended the use of 4 categories (excellent, good, fair, and poor) based on serum IgG concentration and associated calf morbidity, instead of the traditional standard. In the current study, we used 4 categories based on serum γGLB concentration, according to Lombard et al. (2020), and demonstrated that γGLB concentration can be used as a predictive indicator for calf health, similar to serum IgG concentration.

In conclusion, serum γGLB concentration can be used to estimate serum IgG concentration with high precision regardless of whether calves are fed WC or CR. In addition, serum γGLB concentrations in the first week of age are associated with morbidity before weaning in dairy calves. These results suggest that serum γGLB concentration is a predictive indicator for calf health similar to serum IgG concentration.
